# Potential benefit of angiotensin II in COVID-19 patients: beyond reasonable doubt?

**DOI:** 10.1186/s13054-020-03027-w

**Published:** 2020-06-09

**Authors:** António Tralhão, Luís Ferreira Moita, Pedro Póvoa

**Affiliations:** 1 Polyvalent Intensive Care Unit, Hospital de São Francisco Xavier, Centro Hospitalar Lisboa Ocidental, Estrada do Forte do Alto do Duque, 1449–005 Lisbon, Portugal; 2 Cardiology Department, Hospital de Santa Cruz, Centro Hospitalar Lisboa Ocidental, Avenida Professor Doutor Reinaldo dos Santos, 2790–134 Carnaxide, Portugal; 3 Innate Immunity and Inflammation Laboratory, Instituto Gulbenkian de Ciência, Rua da Quinta Grande 6, 2780-156 Oeiras, Portugal; 4 NOVA Medical School, CHRH, New University of Lisbon, 1069–056 Lisbon, Portugal; 5 Center for Clinical Epidemiology and Research Unit of Clinical Epidemiology, OUH Odense University Hospital, DK–5000 Odense C, Denmark

We read with great interest the editorial by Busse et al. on the potential use of angiotensin II in the treatment of COVID-19 [1]. However, amidst the myriad of attempted interventions, some may be more reasonable than others [2].

In preclinical studies, SARS-CoV-1-mediated ACE-2 downregulation led to increased lung injury [3]. The extrapolated protective role of ACE2 in human lung infections seems to result from both the breakdown of angiotensin II and the generation of Ang1–7 and Ang1–9, anti-inflammatory, antioxidative, and antiproliferative peptides that thwart the detrimental effects of angiotensin II in lung parenchyma [3]. Notably, angiotensin II receptor I blockade salvaged a rodent model from more profound alveolar damage [3]. In a clinical setting, recombinant human ACE2 significantly reduced angiotensin II and hinted towards lower mortality in a small randomized trial of undifferentiated ARDS patients [4]. Taken together, these studies cast little doubt on the true colors of angiotensin II during severe COVID-19 pneumonia. Conversely, observational data have revealed no harm and even disclosed potential benefit associated with RAAS modulation [5]. Hence, the assumption of Busse et al. is frankly counterintuitive. Ongoing RCTs evaluating rhACE2 (NCT04335136), AT-1 receptor blockade (NCT04312009), and ACEi/ARB continuation or discontinuation after COVID-19 diagnosis (NCT04329195) are eagerly expected to shed more light into present uncertainties.

Busse et al. advocate for the compassionate use of angiotensin II in critically ill patients with supervening shock and suggest it may even be used prophylactically. In an aged population with cardiovascular comorbidities, in which RAAS blockade has earned a pivotal protective role for decades, such radical shift could have additional unforeseen consequences. Angiotensin II effectively increased blood pressure on top of norepinephrine in the ATHOS-3 trial. Although certainly appealing and offering an alternative pathway to improve mean arterial pressure and organ perfusion in vasodilatory shock, more limb ischemia and de novo infections were also noted, raising safety concerns. In severe COVID-19 patients, in whom the percentage of refractory shock in unclear, the utility of a second vasopressor seems even more unwarranted.

From our perspective, it appears unlikely and even paradoxical to anticipate a net clinical benefit of angiotensin II in COVID-19. If reasonable doubt still persists, this assumption should be put to the test like other putative beneficial interventions [2]. Beyond their individual plausibility, all proposed therapies in COVID-19 patients should be considered experimental and cannot be universally recommended until evaluated in properly conducted RCTs.

## Authors’ response

Angiotensin II for COVID-19-induced shock: beyond a reasonable doubt, an ACE in the hole

Michael T. McCurdy, Jonathan H. Chow, Ashish K. Khanna, and Laurence W. Busse

We thank Tralhão et al. for bringing up important issues regarding our commentary on the use of angiotensin II for vasodilatory shock in COVID-19 patients. Given recent data to support continuing ACE-inhibition in patients with COVID-19, we are not arguing to cease such therapy in hemodynamically stable patients. However, those with vasodilatory shock are clearly not the same as those on ACE-inhibitors, and the “pivotal protective role” of RAAS blockade has been associated with increased risk of hemodynamic compromise in critically ill patients [6]. We also caution against an argument based on the pilot study by Khan et al. of recombinant human ACE2 for patients with ARDS, which was halted due to clinical futility and relies only on angiotensin II levels, rather than the ratio of angiotensin II to angiotensin I, which may have greater clinical relevance [7, 8].

Dr. Tralhão’s argument that lung injury results directly from increased angiotensin II levels due to virally mediated downregulation of ACE-2 rather than from direct viral invasion not only ignores basic human physiology but also runs contrary to the currently available evidence. Had increased levels of angiotensin II been detrimental to lung parenchyma, this would have been suggested by the results of ATHOS-3, which showed no such effect [9]. Further data support the safety of angiotensin II in patients with COVID-19. Zangrillo et al. recently reported using angiotensin II for COVID-19-induced vasodilatory shock in 16 patients, 10 of whom received it as a first-line and only requisite vasopressor [10]. Contrary to concerns expressed by Tralhão et al., patients treated with angiotensin II had significant *improvements* in FiO_2_ (0.70 to 0.40), PEEP (14 to 11 cmH_2_O), and SpO_2_/FiO_2_ ratio (121.4 to 200.0) at 48 h. Despite staggeringly high global mortality rates for COVID-19-induced vasodilatory shock, 14 of the 16 patients in this case series were alive at the time of the authors’ submission of their report.

Cognizant of the dilemma of having to manage critically ill patients in the absence of disease-specific data, we must continue to rely on tangentially-related, randomized controlled trials like ATHOS-3, as well as convincing clinical experience, as provided by Zangrillo et al. We fully support Dr. Tralhão’s suggestion that well-designed studies should inform our treatment options. While some clinicians may lack clinical equipoise regarding angiotensin II, we still maintain that it should be evaluated in the setting of COVID-19, based on a rational physiological argument and emerging supportive data.

## Data Availability

Not applicable.

## Abbreviations

ACE: Angiotensin converting enzyme
ACE2: Angiotensin converting enzyme 2
ACEi: Angiotensin converting enzyme inhibitors
Ang: Angiotensin
ARB: Angiotensin receptor blocker
ARDS: Acute respiratory distress syndrome
AT-1: Angiotensin II receptor I
RAAS: Renin angiotensin aldosterone system
RhACE2: Recombinant human angiotensin converting enzyme 2
RCT: Randomized clinical trial
SARS-Cov-1: Severe acute respiratory syndrome coronavirus 1
